# Sex-specific strategies of resource allocation in response to competition for light in a dioecious plant

**DOI:** 10.1007/s00442-017-3966-5

**Published:** 2017-10-17

**Authors:** Jeanne Tonnabel, Patrice David, John R. Pannell

**Affiliations:** 1 Department of Ecology and Evolution, University of Lausanne, 1015 Lausanne, Switzerland; 2 Centre d’Ecologie Fonctionnelle et Evolutive (CEFE), UMR 5175, CNRS, Université de Montpellier, Université Paul Valéry Montpellier, EPHE, 1919 route de Mende, 34293 Montpellier Cedex 5, France

**Keywords:** Plasticity, Sexual dimorphism, Resource allocation, Light competition, Carbon limitation

## Abstract

The differential plasticity hypothesis suggests that sexual dimorphism in dioecious plants could evolve in response to sex-specific resource requirements for reproduction (i.e., high carbon requirements for ovules and high nitrogen demands for pollen). When resources become limiting during growth, males and females should, therefore, adjust their allocation to resource-harvesting organs differently. To investigate the potential for plants to respond to resource limitation late in life and to test the differential plasticity hypothesis, we grew male and female individuals of the annual wind-pollinated plant *Mercurialis annua* in a common garden. Late in the growth season, we simulated a change in competition by decreasing plant density in half of the replicates. We measured both allocation to vegetative and reproductive traits and analyzed the relative allocation to reproduction vs. growth. Males and females differentially adjusted their resource allocation in response to varying plant densities, despite the fact that they were reproductively mature. Males maintained the same relative allocation of resource to reproductive vs. vegetative tissues at both densities. In contrast, females reduced vegetative growth proportionally less than seed production at the higher density. Our results highlight the dynamic nature of allocation decisions taken by plants, which respond quickly and in a sexually dimorphic way to changes in their competitive circumstances. The existence of resource ‘currencies’ limiting male and female functions differently have potentially led to the evolution of sex-specific strategies of resource acquisition and deployment, with females conserving resources for vegetative organs to ensure their future carbon-rich reproduction.

**Electronic supplementary material:**

The online version of this article (doi:10.1007/s00442-017-3966-5) contains supplementary material, which is available to authorized users.

## Introduction

Most plant species are hermaphroditic, with individuals having both male and female functions within the same flower. In contrast, dioecy is relatively uncommon among flowering plants, with only 4–6% of all angiosperm species having fully separate sexes (Renner and Ricklefs 1995; Renner 2014). Nevertheless, Renner (2014) estimated that transitions from hermaphroditism to dioecy have occurred between 871 and 5000 times during the evolutionary history of angiosperms. Such transitions are often associated with the evolution of sexual dimorphism, probably because males and females maximize their respective fitness by evolving divergent life history traits, possibly allowing for sex-specific resource-acquisition strategies.

Sexual dimorphism has been documented in many plant species for a wide range of phenotypic characters. Males and females typically differ in key aspects of their morphology, physiology, patterns of allocation to life history traits and defense traits (reviewed in Geber et al. 1999; Avila-Sakar and Romanow 2012; Barrett and Hough 2013). Importantly, the degree of sexual dimorphism not only varies among species but also among populations of the same species, both in plants and animals (e.g., between species: Harris and Pannell 2010; Laiolo et al. 2013; Tonnabel et al. 2014; within species: Teder and Tammaru 2005; Delph et al. 2002; Blanckenhorn et al. 2006; Stillwell et al. 2007).

Three main hypotheses have been proposed to explain the evolution of sexual dimorphism: the female-choice hypothesis; the male–male competition hypothesis; and the differential plasticity hypothesis (reviewed in Hedrick and Temeles 1989). As pointed out by Delph and Bell (2008), such hypotheses can also explain intra-specific variation in sexual dimorphism. Female choice probably accounts for many cases of sexual dimorphism in animals, where males compete for the attention of their prospective mates, but the scope for female choice in plants is likely usually limited to the short period of time during which pollen grains grow down a recipient plant’s style (Marshall and Folsom 1991; Lankinen and Madjidian 2011). In contrast, male–male competition is probably frequent in plant populations and important for the evolution of sexually dimorphic characters. Male gametophytes, by being mobile, compete for access to the immobile gametophytes of females, potentially favouring traits that facilitate pollen dispersal (Bond and Midgley 1988; Bond and Maze 1999; Cocucci et al. 2014).

Finally, the differential plasticity hypothesis posits that males and females differ in their plastic response to one or more environmental factors, thus producing variation in the degree of sexual dimorphism along environmental gradients (Delph and Bell 2008). A key idea behind the differential plasticity hypothesis is that males and females might require different resource ‘currencies’ (e.g., carbon, nitrogen, phosphorus) for their reproduction (Ashman and Baker 1992; Obeso 2002). Most sex-allocation thinking in plants implicitly assumes the division of a single common resource currency between male and female functions. However, there is some evidence that seed production (i.e., female function) is mostly limited by water and carbon (Antos and Allen 1990; McDowell et al. 2000), whereas several studies have highlighted the high requirements of nitrogen for pollen production (i.e., male function), particularly in wind-pollinated species that produce large amounts of pollen (Harris and Pannell 2008; Van Drunen and Dorken 2012). Thus, male and female resource-acquisition traits have probably been selected differently, resulting in a sex-specific somatic cost of reproduction for these different resource currencies, i.e., sex-specific trade-offs of resource allocation between reproduction and growth of resource-harvesting organs (Obeso 2002; Barrett and Hough 2013).

Given the likely differences in resource requirements between males and females, one might expect the evolution of sex-specific plasticity in traits affecting resource acquisition in response to the environmental variation in the availability of such resource currencies (i.e., nitrogen and carbon). For instance, females might respond plastically to carbon limitation so as to secure more carbon than males, thus maintaining a capacity for future fruit production. In perennial species and annuals with indeterminate reproduction (in which flower and fruit production occur over most of their growth period), female plants are likely subjected to a subtle compromise between future carbon needs of seeds and a future capacity for carbon acquisition. Age-dependent female resource allocation thus should probably involve allocating carbon to flowers only when they can also allocate sufficient resources towards shoots that are needed later to supply carbon for seed development, a balance that should be struck differently under different levels of competition for light. Here, we evaluate patterns of sexual dimorphism that follow from the differential plasticity hypothesis using an experiment that varied the density of plants during the course of their growing season.

Variation in a plant’s carbon budget can, of course, result from variation in light reception or in water availability, both of which may limit photosynthesis. However, because it has been shown in some species that males express a higher investment into roots than do females, presumably in response to high nitrogen and other nutrient requirements (Harris and Pannell 2008), variation in water availability might affect males and females differently in their respective ability to perform photosynthesis. As far as we are aware, only one study has examined the differential plasticity hypothesis from the point of view of the relative allocation made to reproduction vs. growth (i.e., reproductive effort; Harris and Pannell 2008). Harris and Pannell (2008) focused on variation of nitrogen limitation, i.e., the resource currency that limits male more than female function, but the question of how sex-specific reproductive effort varies in response to carbon limitation (thus potentially limiting especially female reproduction) has to our knowledge not yet been investigated.

Unlike other resource currencies, light has the peculiarity of establishing strongly asymmetrical competition in plants, with the tallest plants removing a disproportionately large amount of the resource (Weiner 1990). It is, therefore, critical for plants to evolve plastic responses to light deprivation: such a plastic response is commonly labeled a ‘shade-avoidance’ response, and is known to occur through the perception of the presence of neighboring biomass by a decrease in the red:far-red (R:FR) light ratio (Schmitt and Wulff 1993; Sleeman et al. 2002). When daylight passes through vegetation, the R:FR ratio is lowered as a result of red absorption by photosynthetic pigments. The shade-avoidance response to this shift in the light spectrum typically involves increase in plant height through internode elongation and decreased branching (Schmitt and Wulff 1993; Sleeman et al. 2002). This greater resource allocation to vertical growth also involves a trade-off giving rise to a decrease of resource allocation towards reproduction (Harper 1977), but it also leads to a decrease in total plant biomass, probably as a result of light limitation (Labouche and Pannell 2016). The shade-avoidance response is well known, but the responsiveness of fully reproductive plants to a sudden change in the level of competition has, to the best of our knowledge, never been investigated. Given that several ecological processes can lead to fast and intense changes in vegetation cover in natural populations (e.g., herbivory of neighbors, colonization of fast-growing plants, or climatic perturbations), we might expect a plastic response to changed resource availability during growth, even after reproduction commences.

Our study presents results of an experiment that aimed at determining whether plants respond dynamically to a change in carbon availability when they are already fully mature, and the extent to which such a response is sexually dimorphic and reflects expectations based on the existence of sex-specific resource currency requirements. We grew males and females of the annual dioecious plant *Mercurialis annua* together at an equal sex ratio, under semi-natural conditions, and assessed the effect on each of the sexes of an imposed change in plant density late during growth and reproduction. We tested the prediction that, to satisfy their needs in carbon for future reproduction, females should respond to increased competition for light by adopting a resource allocation strategy that maximizes their future capacity for carbon acquisition: (1) either by decreasing their resource allocation towards aboveground biomass less than males do; or (2) by decreasing their allocation towards reproductive biomass more than males do. Our experiment highlights the dynamic nature of responses to the intensity of competition for light, even after plants are fully mature. It also provides new evidence in support of the differential plasticity hypothesis, notably in the context of sex-specific resource requirements.

## Materials and methods

### Study species and seed collection

*Mercurialis annua* is a wind-pollinated annual herb inhabiting disturbed habitats in western Europe and around the Mediterranean Basin (Tutin et al. 1964). Populations of *M. annua* display striking variation in their sexual systems, with dioecious, androdioecious and monoecious populations occurring in different parts of its range (Durand 1963; Pannell et al. 2004). In the present study, we focused on dioecious populations of *M. annua*, in which males are typically smaller than females from an early stage in their growth (Harris and Pannell 2008). Males of *M. annua* also produce green staminate flowers that are held on erect ‘peduncles’, whereas females produce green dehiscent subsessile capsules of two or three seeds in their leaf axils. In both males and females, flowering begins several weeks after seeds germinate and continues during plant growth, with new inflorescences produced indeterminately in each new leaf axil over a period of several months (Pannell 1997). Because individuals of *M. annua* still continue to grow and reproduce over an extended period during the flowering season, its life cycle has similarities with that of perennial iteroparous plants, whose reproduction is spread over multiple seasons. In the Mediterranean part of its range, *M. annua* germinates in autumn but performs most of its growth and reproduction in spring. Therefore, in spring, *M. annua* likely suffers from an increase in crowding due to competition with fast-growing species and leaf formation of co-occurring shrubs.

We used a pool of mixed seeds collected from 35 populations from a metapopulation in northern Spain; seeds from approximately 30 females had been sampled from each population. These seeds were initially grown in a common garden setting for three generations (from 2012 to 2014) in Lausanne, Switzerland, to eliminate any potential maternal effects related to, e.g., the population of origin. Growing the plants for several generations in a common environment prior to the experiment also allowed us to partially scramble the genomes originating from different populations.

### Experimental design

To study sex-specific patterns of phenotypic plasticity in response to density, we established an outdoor experiment at the experimental field platform of the LabEx CeMEB in Montpellier, France. The experiment involved two density treatments, with equal sex ratios and five replicates per treatment. Seeds were initially germinated in greenhouses in February 2015, using separate pots in sterile compost. After 7 weeks, when plants had begun flowering and could be sexed, pairs of males and females were transplanted jointly into 2-l pots containing a sterile soil mix (1/3 of sieved clay and chalky soil, 1/3 of recycled compost and 1/3 of compost). To reduce asymmetries in competition caused by slight differences in the timing of germination, we associated in each pot a male and a female of similar height at the onset of the experiment.

Each male–female pair was randomly assigned to one of the ten outdoor experimental populations. Each of our ten experimental populations was formed of 100 male–female pairs, with each pair established in a separate pot. Pots were sunk into the soil to minimize wind damage and to prevent rapid desiccation of soil within the pots. At this first stage, all populations were kept at the same density, corresponding to pots separated by 1.0 m. After 4 weeks, when plants had reached full maturity and had begun to disperse pollen and seeds (while continuing to produce new flowers), the pots in five experimental populations were moved closer together such that their density then corresponded to an inter-pot separation of 20 cm, which previous work (Labouche and Pannell 2016) has shown to elicit a shade-avoidance response. Pots in the low-density treatment were similarly moved, but pot separation was maintained at 1.0 m.

Because our plots were distributed over an experimental field subject to a potential environmental gradient (due to the presence of a slight slope), we adopted a block design for analysis, with each of two blocks containing two or three replicates of each treatment, respectively, (because of space constraints, we could not set up a fully balanced block design). Because all females were paired in the same pot with a male, and likely also because of the large number of males in each array, female reproduction was not pollen limited (see Hesse and Pannell 2011a). Our two contrasted density treatments were applied for a further 4 weeks, a duration chosen to be considerably shorter than the typical reproductive season of *M. annua*. At the end of the 4 weeks when the plants were 14 weeks old, plant allocation to vegetative and reproductive structures was assessed for a random sample of 25 pairs of males and females per experimental population, except for two experimental populations (being studied as part of a separate experiment) in which all individuals were measured. This resulted in a total of 400 pairs of males and females measured. Because plants at the center of the experimental populations might respond to plant density differently than at their edge (Labouche and Pannell 2016), we sampled plants randomly from both edge and center pairs; the likely additional variance contributed by center vs. edge effects probably renders the results of our analysis conservative. Pairs of males and females shared soil in pots for both treatments, so that below-ground competition was similar at both densities. We chose to not only increase rather than decrease density mid-way through the growth season both to ensure good growth conditions for plants early in life but also because effective density likely often increases in natural populations of *M. annua* in spring (i.e., due to competition with fast-growing plants or germination before leaf formation of co-occurring shrubs).

### Growth and reproductive allocation measurements

To assess male and female resource allocation at low versus high densities, we measured traits for both growth and reproduction. We measured allocation to growth and reproduction 12 weeks after the onset of flowering while plants were still growing. We first measured traits in vivo, before harvesting the aboveground portions of the plants, which were bagged and dried. For practical reasons, vegetative and reproductive parts were dissected prior to drying in males, while for females these two components were separated after drying. We also measured plant height, plant diameter, dry biomass and the length of the first two branches, which we later averaged. In males, we assessed plant height by excluding the erect pedunculate inflorescences, which were taller than the highest pair of leaves; this meant that height could be compared directly between males and females. Allocation to reproduction in females was estimated by measuring the number of seeds, the total seed weight and the seed size. The total number of seeds per plant, and relative seed size, were measured using an automatic seed counter (Elmor C3; Elmor Angewandte Elektronik, Schwyz, Switzerland). Finally, allocation to pollen production and dispersal in males was estimated by measuring the total number of inflorescences produced, the total dry mass of flowers, the number of inflorescences displayed above the plant, and the length of the peduncle of the highest inflorescence. The rationale for these two last measurements was that they might reflect some investment in pollen dispersal. Previous studies have shown that weighing the mass of male flowers provides a good estimate of investment in pollen production in *M. annua* (Pannell 1997). Lengths of vegetative traits were measured in centimeters, mass of both vegetative and reproductive organs were assessed in grams, and size of seeds was measured on a relative scale with arbitrary units. Five people were involved in data acquisition, and their measurements were randomly distributed among replicates and treatments.

### Statistical analyses

To test for the effect of plant density on the sex-specific resource allocation to vegetative and reproductive structures, we analyzed linear and generalized mixed models on males and females separately on several traits relevant to growth and reproduction. The analysis was performed for each life history trait independently, with plant density treated as a fixed factor and both block and population as random. Linear mixed models were used for all response variables, except for the number of peduncles displayed by males above the plant, for which we used a generalized linear mixed model with a Poisson error distribution; this latter trait was the only one that did not meet assumptions of normality; here, individual plants were added as a random factor to account for over-dispersion of data. To investigate whether density affected the relative allocation to reproductive vs. vegetative tissues, we calculated the reproductive effort by dividing the total mass of male flowers or seeds by resources allocated to the plant as a whole (considering aboveground parts only). For vegetative traits, that are common to both sexes, we tested the effect of density on sexual dimorphism (equivalent to the interaction between sex and density) by calculating the male–female difference within each pot, and testing the density effect on this difference, to keep the same models and data structure as in the analyses performed separately for each sex. We could not perform such a comparison directly for reproductive traits because male and female reproduction was measured in different units (e.g., seed number for females and inflorescence number for males). However, to investigate our differential plasticity hypothesis further, we calculated for each population and for each sex the relative allocation to vegetative and reproductive biomass as a percentage of change in biomass compared to the mean allocation to these tissues across populations at the low density. All models were validated by checking their error structure graphically. Only reproductive effort was log-transformed to meet assumptions for analysis. The effect of plant density and associated statistical significance were tested using likelihood ratio tests. Data are given as means ± standard errors (SE), and graphical representation of the raw data is provided in Appendices (Figures S1–S3). Because a plastic response in one trait could be the result of a genetic and phenotypic correlation with another trait, we also provide in Table S1 the correlation coefficients between all measured traits separately for males and females. All analyses were conducted in R 3.2.2 (R Core Team 2015) using the package lme4 (Bates et al. 2014) to implement linear and generalized linear mixed models.

## Results

### Sexual dimorphism in vegetative traits

At both densities, males displayed lower investment in all vegetative traits compared to females, as shown by the respective distribution of trait values for males and females, respectively, in: plant height (41.4 ± 0.37 cm vs. 45.3 ± 0.37 cm); biomass (2.34 ± 0.04 g vs. 4.28 ± 0.08 g); mean length of the first branches (7.80 ± 0.20 cm vs. 8.87 ± 0.23 cm); and plant diameter (9.65 ± 0.13 cm vs. 12.3 ± 0.16 cm). Reproductive effort was greater in males than in females at both densities (0.20 ± 0.003 vs. 0.11 ± 0.002). Both males and females showed moderate positive correlations between traits representing allocation to growth (i.e., highest coefficient of correlation was 0.43 and 0.56, respectively, Table S1a, b). Correlations between reproductive and vegetative traits were also moderate and positive in both males and females (i.e., highest coefficient of correlation was 0.75 and 0.70, respectively, Table S1a, b).

### Sex-specific plastic response to density

**Fig. 1:**
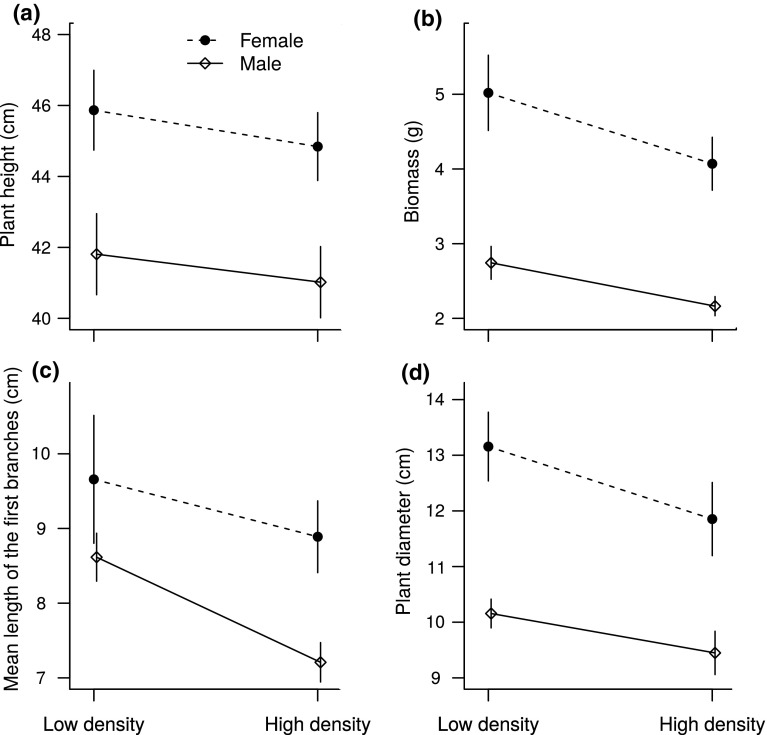
Effect of density on allocation to vegetative tissues for both males and females of *M. annua* grown in a common garden. Data are given as means across populations ± SE

In males, both plant biomass and the mean length of the first branches were lower for the high-density treatment, as seen by a significant effect of density (Table 1, Fig. 1b, c). In males, plant height and diameter tended to be lower at the high compared to the low density (Fig. 1a, d), but this tendency was not significant (Table 1). In females, all vegetative traits showed a non-significant tendency to be lower at the higher density (Table 1, Fig. 1a–d), and the difference in plant diameter and biomass were only marginally significant. However, differences in resource allocation to vegetative growth between males and females, calculated as differences per pot, did not display a density effect for any of the four vegetative traits measured (Table 1).

**Table 1:** Results of the ANOVAs for the effect of density (low vs. high) on male and female traits of *M. annua* grown at contrasted density in a common garden, and on the difference between male and female traits within each pot

Traits	Effect of density on trait values						Effect of density on difference between sexes	
	Females			Males			*χ* ^*2*^	*p* value
	*β*	*χ* ^2^	*p* value	*β*	*χ* ^2^	*p* value		
Vegetative traits								
Plant height	−0.46	0.12	0.73	−0.36	0.06	0.81	1.30	0.25
Plant biomass	−0.66	3.60	0.06	−**0.40**	**9.43**	**0.002**	0.91	0.34
Mean length of the first branches	−0.74	1.04	0.31	−**1.74**	**9.53**	**0.002**	0.30	0.58
Plant diameter	−0.97	3.77	0.05	−0.38	2.10	0.15	2.58	0.11
Reproductive traits								
Total seed weight	−**0.13**	**6.35**	**0.01**					
Mean seed size	−**2.26**	**4.38**	**0.04**					
Seed number	−**33.14**	**6.28**	**0.01**					
Total inflorescence weight				−**0.07**	**10.98**	**0.0009**		
Total number of inflorescences				−**11.80**	**9.72**	**0.002**		
Length of the higher peduncle				−**0.38**	**9.09**	**0.003**		
Number of peduncles above the plant				−0.07	0.92	0.34		
Relative allocation to vegetative and reproductive traits								
Total seed weight/plant biomass	−**0.10**	**6.36**	**0.01**					
Total peduncle weight/plant biomass				−0.01	0.14	0.70		

*β* indicates the value of the linear coefficient for plant density. *β*, *χ* ^2^ and *p* value are in bold when the *p* value < 0.05 (ddl = 1)

**Fig. 2:**
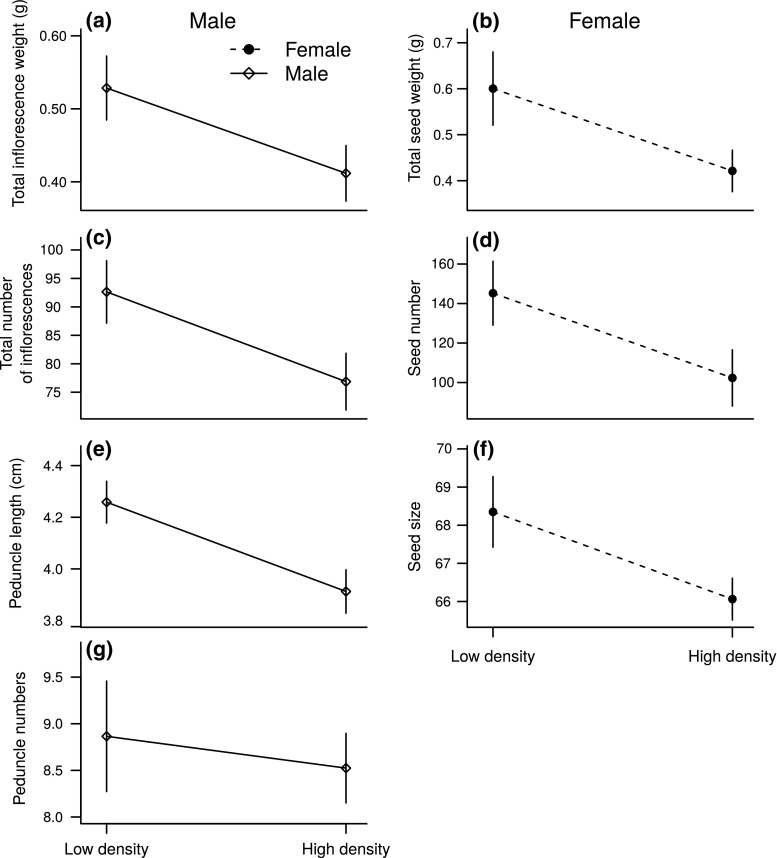
Effect of density on allocation to reproductive tissues for both males and females of *M. annua* grown in a common garden. Data are given as means across populations ± SE. Seed size is a relative scale with no unit. Peduncle length corresponds to the length of the highest peduncle and peduncle numbers to the number of peduncles displayed above the plant

In both males and females, all reproductive traits measured (males: mass of flowers, total number of inflorescences; females: total seed weight, seed number, seed size), were significantly reduced at the higher density (Table 1, Fig. 2a–d, f). In males, traits reflecting allocation towards pollen dispersal displayed contrasting results in response to plant density: the length of the highest peduncle was significantly lower at the higher density (Table 1, Fig. 2e), but the number of peduncles above the plant remained unchanged between densities (Table 1, Fig. 2g).

### Relative resource allocation to vegetative growth vs. reproduction

**Fig. 3:**
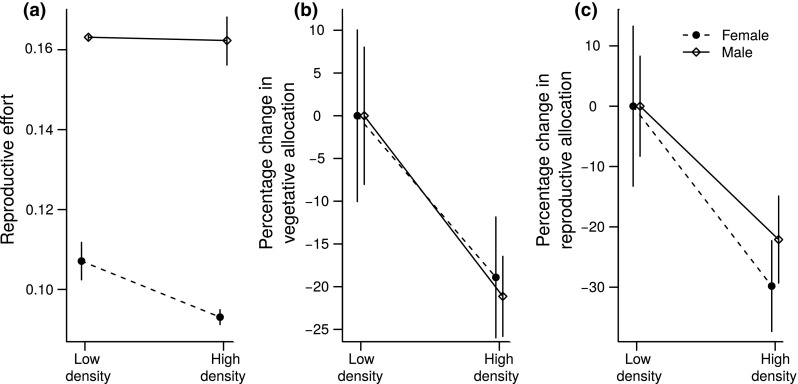
Effect of density on reproductive effort and allocation to vegetative and reproductive tissues relative to their average allocation; values are means across population (± SE) at low density, for males and females of *M. annua* grown in a common garden. **a** Reproductive effort (inflorescence and seed weight divided by total biomass); **b** vegetative and **c** reproductive allocations are calculated as percentage change in biomass of the respective tissues compared to the mean allocation to these tissues across populations at low density

Only females displayed a plastic response in their relative allocation to reproductive vs. vegetative tissues (i.e., reproductive effort): males maintained the same reproductive effort, whereas this ratio was significantly lower at the higher density for females (Table 1, Fig. 3a). An increase in density thus caused females to reduce their relative allocation to reproduction more than males did. Vegetative biomass tended to decrease less in females than in males at the higher density (Fig. 3b), while allocation towards reproductive biomass at the higher density tended to decrease more in females than in males (Fig. 3c).

## Discussion

Overall, our study found that plants of *M. annua* display considerable flexibility in resource allocation in response to local competition conditions. Over the course of only 4 weeks, reproductively mature males and females differed in how they adjusted their relative resource allocation to vegetative and reproductive tissues in response to altered levels of aboveground competition. While males maintained the same relative allocation of resources to reproductive vs. vegetative tissues, females decreased their relative allocation to reproduction. This points to the existence of a greater trade-off in females than males between current and future reproduction in terms of carbon: in the face of light limitation, a female’s strategy involves decreasing its reproductive effort more than males, probably as a way to safe-guard the future capacity of carbon-harvesting structures for future reproduction and the maturation of seeds. Our results thus conform to the expectations arising from the differential plasticity hypothesis, namely that females should maximize their allocation to aboveground growth under conditions of light limitation more than should males because carbon acquisition is more critical for female reproduction.

### Flexible resource allocation and change in density

Our experiment revealed that resource allocation in *M. annua* is responsive to changes in environmental conditions during the course of plant growth, with reproductively mature plants changing their allocation of resources following a sudden change in aboveground density. Both vegetative and reproductive traits tended to decrease at the higher density of our experiment, reflecting a negative effect of light limitation. However, a plant’s plastic response to the shift in density resulted in reduced allocation to certain traits more than others. Only canopy growth in width and aboveground biomass decreased at the higher density in males. Our results provide new insight on the classical ‘shade-avoidance’ response, which typically includes stem elongation and an increase in plant height (Schmitt and Wulff 1993; Sleeman et al. 2002). The difference between our results and the classical shade-avoidance response (e.g., no difference in plant height) suggests that a plant’s plastic response to a change in density may also vary with plant age and also perhaps with the duration of the change in density.

Studies examining plastic responses in plant growth and allocation have typically applied their treatments when plants are still very young, and little is known about how plants respond plastically to light limitation after they have committed to reproduction, with the exception of studies emphasizing plastic responses of plants to pathogen or predation attacks (reviewed in Heil 2010). Such studies, along with ours, underscore the flexibility of plants in how they allocate resources, and are likely fostered by their modular growth habit, which allows them to respond anew with each additional shoot or inflorescence they produce (reviewed in Schultz et al. 2013).

### The differential plasticity hypothesis in plants

The fact that males and females display different adjustments of their resource allocation to vegetative and reproductive organs with a change in the level of competition for light supports the differential plasticity hypothesis and provides a possible explanation for observed intra-specific variation in sexual dimorphism. Indeed, spatial and temporal environmental heterogeneity can bring about the sort of variation in access to light that we manipulated in our experiment (e.g., different levels of inter-specific competition, predation, perturbations, etc.). More specifically, an increase in density during a plant’s life, as reflected in our treatments, could be caused by fast-growing species in competition with one another, and by canopy closure by competing shrubs and trees after winter. If, as we highlight, resource limitation elicits sex-specific plastic responses, then spatial variation in this resource will lead to spatial variation in the degree of sexual dimorphism or spatial segregation of the sexes (Delph and Bell 2008). Our results add to a growing body of work pointing to the role of differential plasticity in contributing to variation in sexual dimorphism in plants. Most studies that allow investigation of the differential plasticity hypothesis provide support for its importance (Table 2), though the details of how resource currencies are differently reallocated from sources to sinks are still mostly unknown.

**Table 2:** Summary of the experimental studies investigating the differential plasticity hypothesis and providing the name of the species studied, the treatment applied and whether or not a support for differential plasticity was displayed in the study (yes or no)

References	Species name	Treatment variation	Differential plasticity
Conn and Blum (1981)	*Rumex hastatulus*	Plant density and nutrient availability	Yes
Zimmerman and Lechowicz (1982)	*Rumex acetosella*	Water availability	Yes
Lovett-Doust et al. (1987)	*Silene alba*	Plant density	Yes
Gehring and Linhart (1993)	*Silene latifolia*	Light, water, nitrogen, phosphorus, and potassium availability	No
Dorken and Barrett (2004)	*Sagittaria latifolia*	Nitrogen availability	Yes
Delph and Bell (2008)	*Silene latifolia*	Water availability	No
Harris and Pannell (2008)	*Mercurialis annua*	Nitrogen availability	Yes
Herlihy and Delph (2009)	*Silene latifolia*	Water and nutrient availability	Yes
Hesse and Pannell (2011b)		Gender of the competitor and nitrogen availability	Yes
Sánchez Vilas and Pannell (2012)	*Mercurialis annua*	Intra-species and interspecies competition	Yes
Cheng et al. (2014)	*Populus cathayana*	Nitrogen availability	Yes
Labouche and Pannell (2016)	*Mercurialis annua*	Plant density	Yes
Teitel et al. (2016)	*Rumex hastatulus*	Nitrogen availability	Yes

### Light limitation and sex-specific resource trade-offs

We found that females decreased their reproductive effort following an increase in plant density, whereas males kept their reproductive effort constant. This pattern results from a combination of a stronger reduction of allocation towards reproductive tissues in females than in males and a smaller reduction of allocation towards vegetative tissues in females than in males when competition for light increases. This response may be a consequence of a difference in the resource currencies that limit male versus female functions, e.g. carbon may limit reproduction by females more than that by males (Antos and Allen 1990; McDowell et al. 2000). One might expect that a decrease in carbon availability should lead to a smaller decrease in allocation to the carbon-harvesting organs (i.e., shoot and leaves) in females than in males because of the need for females to maintain a capacity for carbon acquisition for later seed and fruit production. If males and females maintain the same allocation towards reproduction, then the sex that incurs the greater cost in carbon, say, would also be subject to a greater somatic cost of reproduction for carbon, and thus decrease allocation to vegetative carbon-rich biomass more than the other sex. Both of these two alternative strategies reflect sex-specific trade-offs between growth and reproduction for different resource currencies. Our results support the first idea, suggesting that the sex incurring the greater cost should invest in organs harvesting the particular resource currency currently limiting its reproduction, in combination with a reduction of allocation to current reproduction. Note that the decrease in reproductive effort observed in females but not in males could partly reflect direct impacts of carbon limitation on the production of carbon-rich female reproductive organs, rather than a specific strategy. However, regardless of proximate causes, the effect of sex-specific reactions to density is that females preserve relatively more capacity for future carbon production than males, thus spreading their reproductive effort in time, when in competition. Our results suggested a higher fraction of biomass devoted to reproductive organs in males than in females, potentially inconsistent with the idea of a higher total carbon cost for female reproduction. However, we did not account for the full investment of females towards their reproduction, as dehiscent capsules were not included in the allocation assay.

Among the previous studies supporting the differential plasticity hypothesis (Table 2), several pointed to the existence of sex-specific carbon budgets consistent with our results. For instance, Lovett-Doust et al. (1987) and Sánchez Vilas and Pannell (2012) showed that females decreased their allocation to growth in response to aboveground competition less than did males. Conversely, other studies report results not conforming to the expectation that females should decrease aboveground biomass less than males when carbon becomes limited. Hesse and Pannell (2011a) reported no strong effect of the level of competition on disparities between males and females on aboveground allocation, and Conn and Blum (1981) and Labouche and Pannell (2016) presented results that do not conform to expectations on the basis of a sex-specific somatic cost of carbon. The divergence between Labouche and Pannell’s (2016) results and ours here probably reflects the fact that, in the former study, competition for light was probably more intense, so that both sexes responded to strong ecological pressure to maintain resource allocation to aboveground biomass (therefore obscuring the expression of sex-specific resource allocation). Our study thus adds to a growing body of work that so far provides mixed support for a stronger somatic cost of carbon in females, potentially responsible for a sex-specific plastic response to carbon limitation.

### Resource allocation and pollen dispersal strategies

In contrast to plant biomass and plant width, we found that both males and females maintained allocation to height at both plant densities. Another important factor potentially resulting in sex-specific selective gradients on plastic responses is the effect of density on pollen dispersal abilities, which may have particularly important implications for the male fitness. In wind-pollinated plants, size may have a direct effect on male fitness, with taller males able to disperse their pollen more effectively and thus enjoying greater siring success (Klinkhamer et al. 1997; and see Eppley and Pannell 2007; Pickup and Barrett 2012 for some experimental evidence). Increased intra-specific densities might provide a signal for increased competition for siring success, and increased inter-specific densities might foreshadow future adverse conditions for pollen dispersal. In addition to sex-specific resource requirement, and assuming that increased plant height indeed enhances male competitive ability for siring seeds, males and females may both have experienced selection to maintain their allocation to plant size in response to a change in plant density: males should benefit from being tall by dispersing pollen from a greater height; and tall females would reduce the risk of losing in the competitive battle for light, an important risk given the extent to which carbon is required for reproduction. Thus, our finding of an absence in sexual dimorphism in plant height across density treatments might result from a combination of sexual selection for a maintenance of plant height in males (for pollen dispersal), and selection for adequate resource acquisition (specifically carbon) in females. In our experiment, we manipulated intra-specific density, increasing at the same time male–male competition for mates and competition for light. It would be interesting to know if these two modes of competition can be uncoupled. One previous study has shown that plastic responses in vegetative growth to density depended on the identity of the competitor (i.e. conspecific or not; Sánchez-Vilas et al. 2011), but future investigations could address its effect on plant reproductive effort and the possible existence of mechanisms of intra- vs. inter-specific competition recognition. Alternatively, a potentially recent transition to dioecy in *M. annua* might explain the absence of sexual dimorphism in response to increased density in plant height, although the relationship between age of separate sexes and degree of sexual dimorphism still remains to be investigated.

## Concluding remarks

Many arguments concerning the evolution of dioecy revolve around genetic and reproductive issues such as the avoidance of self-fertilization and inbreeding depression (Bawa 1980; Charlesworth 1999; Geber et al. 1999). Our results point to the notion that male and female components of reproduction are optimized by different strategies of resource allocation during plant growth. Resource limitation and aboveground competition may sometimes favor dioecy simply because maintaining female fitness and male fitness together becomes inefficient (Charnov et al. 1976). Indeed, light limitation might have led plants to choose between maintaining carbon-harvesting structures for the sake of seed production, or to sacrifice them for the sake of root and male reproduction. Thus, plant competition may produce ‘concave’ fitness trade-off functions and favor dioecy (Charnov 1982; de Jong and Klinkhamer 2005), not only because of purely reproductive interactions (e.g., variation in competition for siring success) but also through dynamics of resource allocation. Our results thus underscore the need to include several resource currencies in models of the evolution of sexual systems and sexual dimorphism in plants, as well as their different sources and sinks.

## Electronic supplementary material

Below is the link to the electronic supplementary material.

Supplementary material 1 (PDF 53 kb)
