# Supplementary material for: Sex-specific strategies of resource allocation in response to competition for light in a dioecious plant
Source: Oecologia. 2017 Oct 17;185(4):675–86. doi: 10.1007/s00442-017-3966-5 (PMC5681607; doi:10.1007/s00442-017-3966-5)
Supplement: Supplementary file 1 — Supplementary material 1 (PDF 53 kb) [file 442_2017_3966_MOESM1_ESM.pdf]

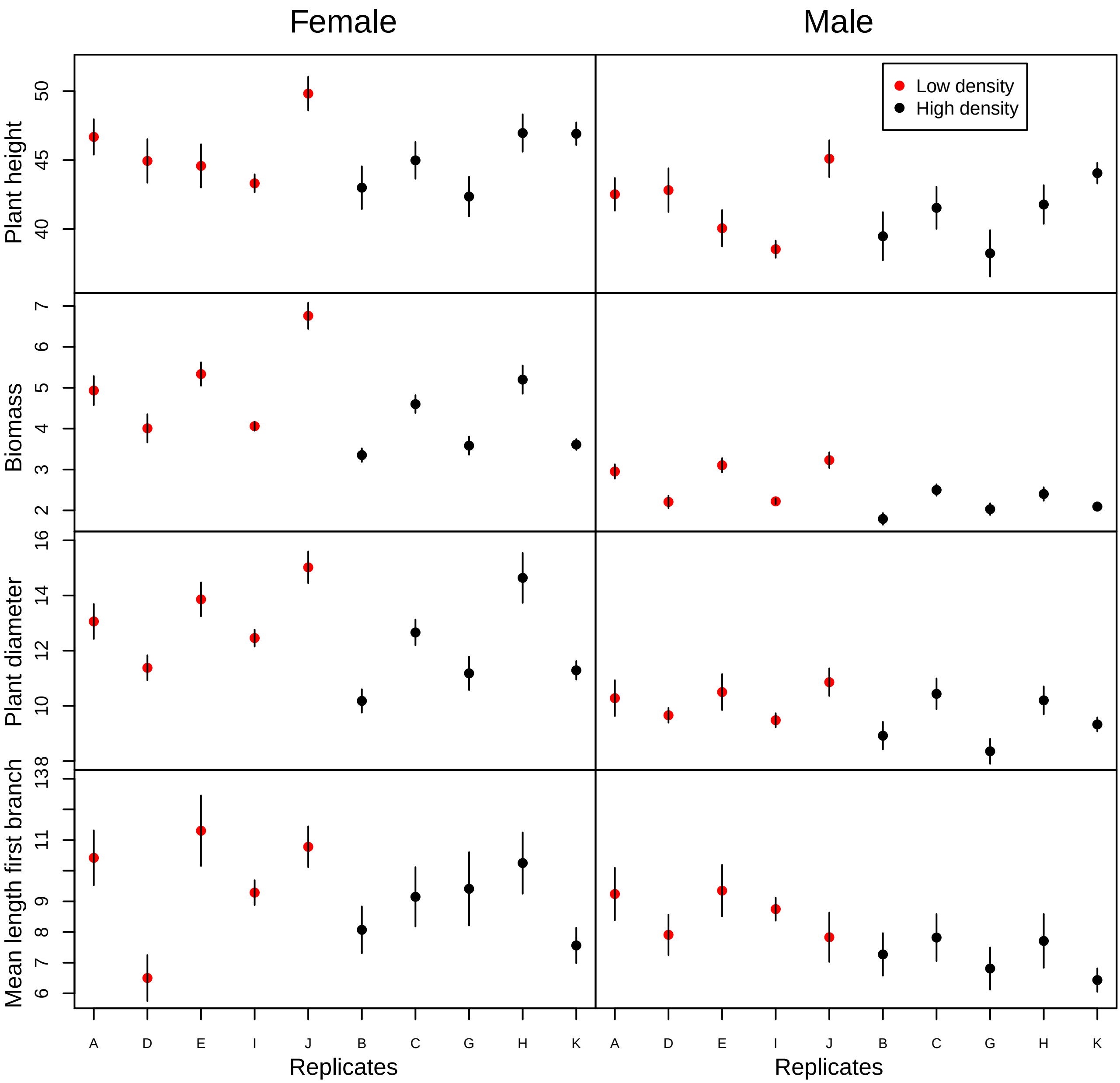

Figure S1: Results on vegetative traits as a function of replicates. Data are given as means per replicate population ± SE. Red and black dots represent respectively low and high densities.

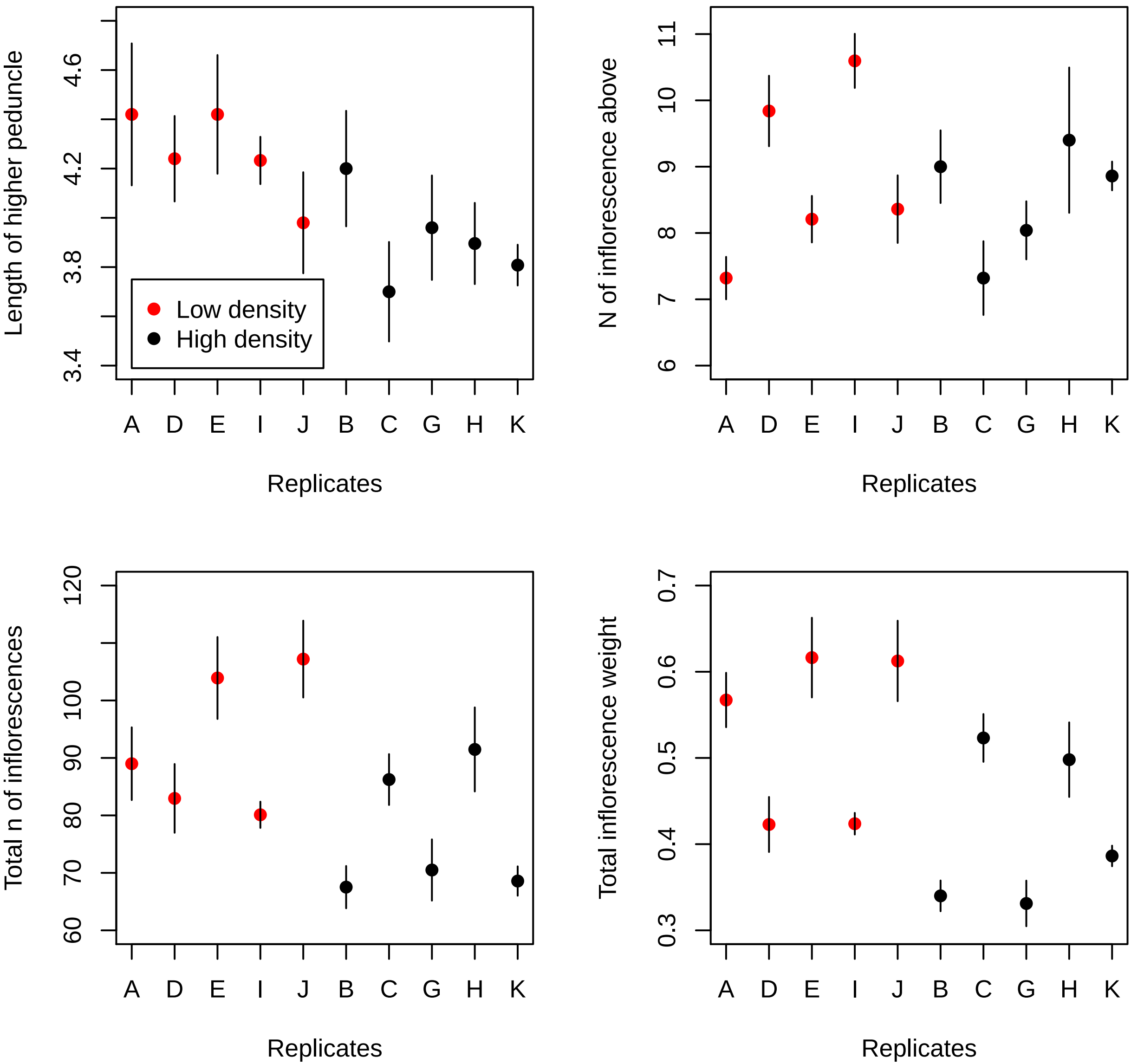

Figure S2: Results on reproductive traits in males as a function of replicates. Data are given as means per replicate population ± SE. Abbreviation: n = number. Red and black dots represent respectively low and high densities.

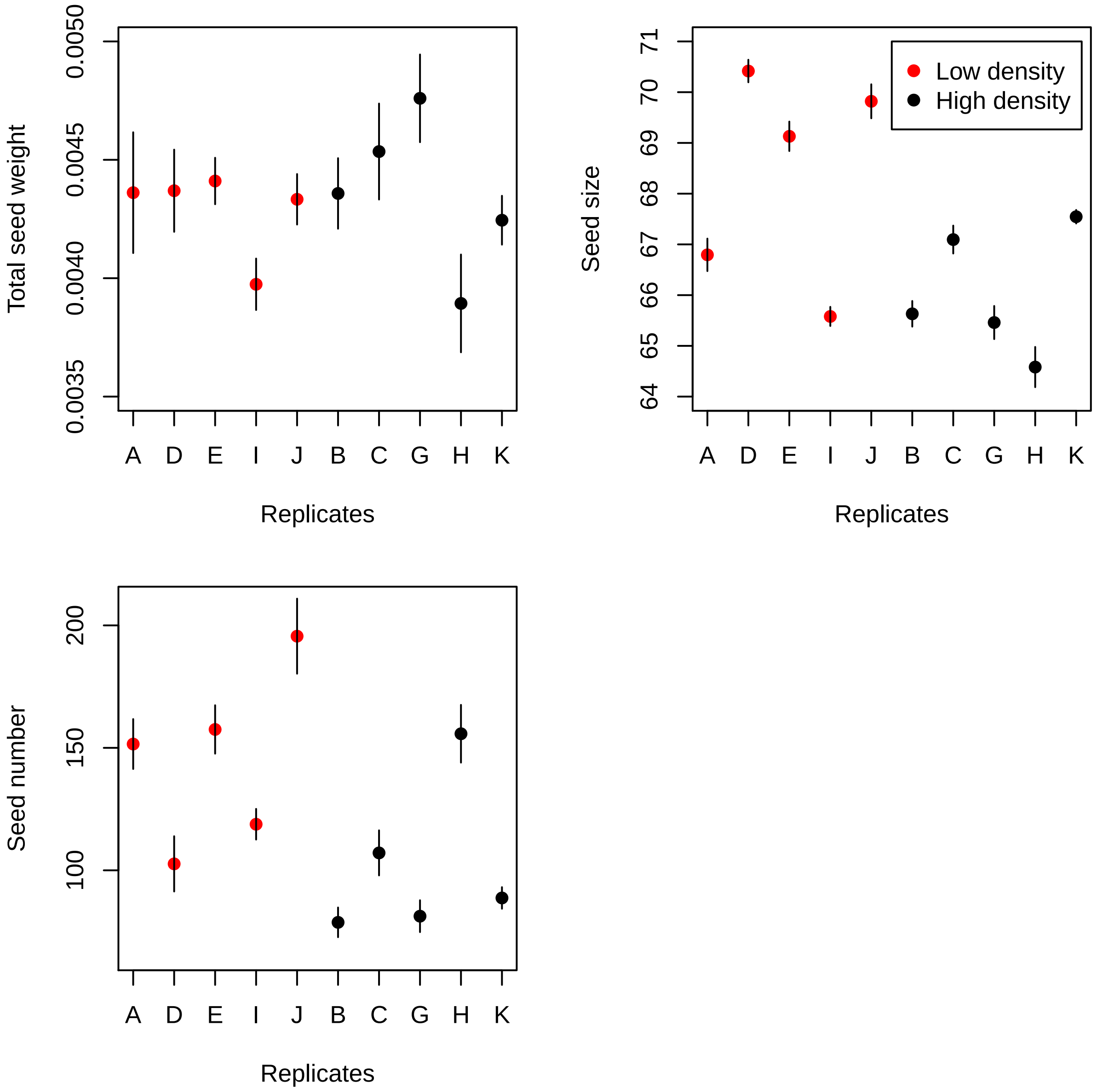

Figure S3: Results on reproductive traits in females as a function of replicates. Data are given as means per replicate population ± SE. Red and black dots represent respectively low and high densities.

Table S1 : Coefficient of correlation between all traits measured in males and females separately (a and b respectivelly). Darker green colour indicates stronger values of correlation coefficients : we have discretized to provide ranges of 0.10 amplitude for each colour. Abbreviation : nb. = number.

a) Males	Plant height	Mean lenght of the first ramification	Plant diameter	Plant biomass	Lenght of the higher pedoncule	Nb. of pedoncules above	Total nb. of inflorescences
Plant height	1.00	-0.24	-0.05	0.26	-0.08	-0.06	-0.01
Mean lenght of the first ramification		1.00	0.49	0.36	0.04	-0.05	0.57
Plant diameter			1.00	0.43	0.09	-0.01	0.45
Plant biomass				1.00	0.06	0.06	0.75
Lenght of the higher pedoncule					1.00	0.30	-0.10
Nb. of pedoncules above						1.00	0.01
Total nb. of inflorescences							1.00
Total flower weight							

b) Females	Plant height	Mean lenght of the first ramification	Plant diameter	Plant biomass	Total seed weight	Seed number	Seed size
Plant height	1.00	-0.06	0.24	0.44	0.28	0.28	0.05
Mean lenght of the first ramification		1.00	0.39	0.37	0.38	0.41	-0.17
Plant diameter			1.00	0.56	0.54	0.56	-0.07
Plant biomass				1.00	0.70	0.67	0.15
Total seed weight					1.00	0.93	0.10
Seed number						1.00	0.00
Seed size							1.00
